# Development of Vaccines against Emerging Mosquito-Vectored Arbovirus Infections

**DOI:** 10.3390/vaccines12010087

**Published:** 2024-01-15

**Authors:** Nicola Principi, Susanna Esposito

**Affiliations:** 1 Università degli Studi di Milano, 20122 Milan, Italy; nicola.principi@unimi.it; 2 Pediatric Clinic, Department of Medicine and Surgery, University of Parma, 43126 Parma, Italy

**Keywords:** arbovirus infection, Chikungunya virus disease, dengue, mosquito, vaccines, West Nile virus disease

## Abstract

Among emergent climate-sensitive infectious diseases, some mosquito-vectored arbovirus infections have epidemiological, social, and economic effects. Dengue virus (DENV), West Nile virus (WNV), and Chikungunya virus (CHIKV) disease, previously common only in the tropics, currently pose a major risk to global health and are expected to expand dramatically in the near future if adequate containment measures are not implemented. The lack of safe and effective vaccines is critical as it seems likely that emerging mosquito-vectored arbovirus infections will be con-trolled only when effective and safe vaccines against each of these infections become available. This paper discusses the clinical characteristics of DENV, WNV, and CHIKV infections and the state of development of vaccines against these viruses. An ideal vaccine should be able to evoke with a single administration a prompt activation of B and T cells, adequate concentrations of protecting/neutralizing antibodies, and the creation of a strong immune memory capable of triggering an effective secondary antibody response after new infection with a wild-type and/or mutated infectious agent. Moreover, the vaccine should be well tolerated, safe, easily administrated, cost-effective, and widely available throughout the world. However, the development of vaccines against emerging mosquito-vectored arbovirus diseases is far from being satisfactory, and it seems likely that it will take many years before effective and safe vaccines for all these infections are made available worldwide.

## 1. Introduction

Due to uncontrolled human activities in the last 50 years, mainly the emissions of greenhouse gases, significant climate changes have occurred in every region of the world [1,2,3]. The average global temperature has progressively increased and is associated with changes in rainfall patterns, increased evaporation, melting glaciers, and rising sea levels. The impact of climate changes on human health has been dramatic and even greater than initially expected [4,5]. Heat-related deaths have increased; air quality has worsened, leading to an increased number of respiratory and cardiovascular diseases; the availability of safe food and drinking water has been reduced with an increased number and severity of gastrointestinal problems; and mental health problems such as depression and post-traumatic stress disorder have become significantly more common or have worsened when already present [6]. Finally, all cli-mate-sensitive infectious diseases have become more common, frequently involving geographic areas that previously were only slightly affected or not affected at all [7]. 

Among emergent climate-sensitive infectious diseases, some mosquito-vectored arbovirus infections have an epidemiological, social, and economic effect. Dengue virus (DENV), West Nile virus (WNV), and Chikungunya virus (CHIKV) disease, previously common only in the tropics, currently pose a major risk to global health and are expected to expand dramatically in the near future if adequate containment measures are not implemented [8]. A suitable example in this regard is the emergence of mosquito-vectored arboviral diseases in some European countries and parts of the USA. This phenomenon is strictly related to the deep alterations that climate changes have made to mosquito life cycles, reproduction, and feeding, extending the areas in which these insects thrive and favoring their re-emergence in areas where they have subsided for decades [9]. Unfortunately, the human response to climate changes and mosquito invasions was initially poor in most of the countries previously not involved, showing that current prevention strategies are not enough to reduce the ongoing emerging viral disease burden. Attempts to eliminate mosquitos were made by implementing traditional disinfestation measures without taking into account the presence of mosquitoes that are resistant to traditional insecticides [10]. Moreover, only recently, the use of mosquito control methods for making genetic or microbial-based alterations to mosquito populations has been considered [11]. The clinical relevance of these infections has been underestimated. The development of antivirals has proceeded slowly [12], as has that of vaccines [13]. 

The lack of safe and effective vaccines is critical as it seems likely that emerging mosquito-vectored arbovirus infections will be controlled only when such vaccines against each of these infections become available. Immunization is considered the most effective approach to preventing and controlling viral infections, as the recent COVID-19 pandemic has clearly demonstrated [14]. This paper discusses the clinical characteristics of DENV, WNV, and CHIKV infections and the state of development of vaccines against these viruses.

## 2. Dengue Virus Disease

DENV is considered to be the most rapidly spreading mosquito-vectored human viral infection in the world. It has been calculated that in 2023, and as of 23 August of that year, over 3.7 million cases had occurred worldwide [15]. Although many patients are asymptomatic and the majority develop mild or moderate signs and symptoms (fever, headache, retroorbital pain, myalgias, arthralgias, nausea, vomiting, and rash or petechiae) that disappear within a few days, about 5% of the cases develop a severe disease, with shock, hemorrhage, and secondary infections that, when not promptly recognized and adequately treated, can lead to death [16]. In the last year, over 2000 DENV-related deaths have been reported from 70 countries/territories globally [15]. 

For years, it has been known that DENV is mainly vectored by Aedes aegypti and Aedes albopictus mosquitoes which, by biting an infected human, can become infected and able to transmit the disease to another human, thereby maintaining the cycle of transmission. DENV infection is endemic in Central and Southern America, Africa, the Middle East, and the Asia–Pacific, where outbreaks occur annually [15]. However, the incidence of DENV infection has increased 30-fold over the last 50 years. Moreover, the disease has appeared in geographic areas where previously only imported cases had been diagnosed. Since 2022, *A. aegypti* has been established in Cyprus and continues to spread to other European countries. *A. albopictus* has been identified in northern and western European countries where in the past it could not survive [17]. In France, in 2022 by 21 October, 65 autochthonous cases were diagnosed, exceeding the total number of cases observed during the entire period 2010 to 2021 [18]. In Italy, in 2020, five locally acquired cases of DENV were detected in a family cluster in the province of Vicenza [19]. In 2023, as of September 18, there were 27 confirmed cases of DENV dis-ease transmitted locally in Italy, of which 21 occurred in a northern region (Lombardy) and 6 around Rome [20]. A similar increase in DENV disease occurrence has been reported in the Americas. In Brazil, the disease is expanding to southern regions that were once off-limits to *A. aegypti* [21]. The same has occurred in Mexico, where this mosquito species was for the first time detected in Mexico City, at an altitude of 2240 m too high to allow mosquitos survive some years ago [22]. Autochthonous cases have also been reported in the USA, such as in Florida, where 10 locally trans-mitted cases were reported in 2023 [23]. 

Four genetically distinct but closely related DENV infections exist [24]. The infection with one DENV serotype usually provides life-long immunity to the same serotype. Unfortunately, it ensures only short-term (1–2 years) protection against infections due to different DENV serotypes. Consequently, an effective vaccine should include all the four DENV serotypes. Several DENV vaccines, based on different platforms but intended to protect from all DENV serotypes, have been developed. However, the development of some of them has been discontinued or is very slow. This is the case for a live attenuated vaccine (TDEN-LAV) whose development has been discontinued [25], an inactivated adjuvanted vaccine (TDEN-PIV) [26], a DNA vaccine [27] for which no recent reports are available, and, finally, a recombinant subunit vaccine (V180) [28] and a live attenuated vaccine (DENV-1-LVHC) [29] whose development has been stopped at phase I. Only three DENV vaccines have reached an advanced phase of development and two of them, Dengvaxia and Qdenga, have been authorized for use in some countries [20]. 

Dengvaxia is a live attenuated vaccine based on the yellow fever virus in which the sequences encoding the pre-membrane (prM) and envelope (E) proteins are re-placed with those coding for the homologous sequences of DENV serotypes 1, 2, 3, and 4 [30]. It has been initially authorized for use in humans in 21 DENV-endemic countries [30]. However, in 2018, Malaysia declined to renew a two-year provisional li-cense, and the Philippines revoked the license as of February 2019. Dengvaxia has also been licensed in Europe and the USA, albeit with differences. In Europe, the vaccine is intended for individuals who are 6 to 45 years of age [31], whereas in the USA, it is authorized for individuals aged 6 to 16 years [32]. In both cases, authorization regards only subjects with laboratory-confirmed previous DENV infection who are living in endemic areas. A schedule of administration comprising three doses 6 months apart is recommended. Restrictions are based on the evidence that vaccine administration to unprimed individuals of all ages, particularly younger children, is poorly effective and increases the risk of severe DENV disease in cases of infection, due to the development of the antibody-dependent enhancement phenomenon (ADE). Dengvaxia elicits both neutralizing and non-neutralizing antibodies, with the highest response for DENV2. Antibodies (Abs) against the pre-membrane (prM) and fusion loop epitope (FLE) of DENV are generally cross-reactive among DENV serotypes but they have suboptimal neutralizing characteristics. By contrast, antibody responses directed against the host cell receptor-binding domain of DENV envelope domain III (EDIII) exhibit a higher degree of type specificity with lower potential for ADE. Suboptimal levels of neutralizing antibodies facilitate the binding of non-neutralizing antibodies to the virus, thus favoring, in the case of infection, DENV entry into monocytes and macrophages, with increased virus replication and risk of severe disease development [33,34]. 

Some clinical trials have evaluated the efficacy and safety of the vaccine in hu-mans, particularly children. Two phase III trials were conducted in the Asia–Pacific region [35] and in Latin America [36] involving, respectively, 10,275 children aged 2–14 years and 20,854 children aged 9–16 years who were monitored through active surveillance until 25 months after randomization. Pooled vaccine efficacy was 60.3% against symptomatic disease by any serotype, with the highest efficacy for serotype 4 (up to 80%) and the lowest for serotype 2 (as low as 33%). An acceptable profile of safety and tolerability was demonstrated, with an incidence of severe adverse events in the period immediately following vaccine administration that was very low (1%) and quite similar to that found in controls. However, whereas efficacy was satisfactory in subjects already infected with DENV (up to 83.7%), it was markedly reduced in sero-negative children, particularly those aged 2–9 years, for whom efficacy was only 14.4%. Moreover, it was calculated that in seropositive participants aged ≥9 years, protection was maintained up to six years after the first dose. Persistence of efficacy was also observed in seropositive participants aged 6–8 years. By contrast, among seronegative participants, the cumulative 5-year incidence of hospitalization for virologically con-firmed DENV disease was higher than in seropositive children, particularly among the youngest, confirming that in seronegative children, this vaccine could be associated with the development of ADE in cases of new DENV infection [37]. This problem, the relatively low efficacy in younger children, the high cost, and the need for three doses to obtain protection explain why this vaccine was not successful even in countries with a high incidence of DENV disease. 

Qdenga is a live attenuated vaccine that consists of an attenuated DENV2 strain and three chimeric viruses expressing the prM and E genes of DENV1, DENV3, and DENV4 using the DENV2 genetic backbone [38]. The use of a backbone of DENV2 instead of yellow fever virus has the potential to stimulate a broader humoral and cell-mediated immunological reaction, avoiding the risk of ADE development. Consequently, it overcomes the limitations previously discussed for Dengvaxia and can be used to vaccinate seronegative subjects and a broader age group including young children [38]. The schedule of administration comprises two doses 3 months apart. Qdenga is authorized for use in subjects older than 4 years in several countries including those in the European Union, the UK, Brazil, Argentina, Indonesia, and Thailand [39]. Immunological studies have shown that this vaccine evokes antibody responses against all four DENV serotypes, albeit with different levels, the highest of which is DENV2. The neutralizing antibody levels are higher in already seropositive subjects but remain above the limit for seropositivity for several years in most individuals. However, due to a lack of knowledge of the serologic correlate of protection, it is not possible to state how long vaccinated subjects remain protected. Efficacy has been evaluated only in subjects aged <16 years, mainly through a phase III clinical trial carried out in Latin America and Asia in which a total of about 20,000 children aged 4–16 years were enrolled. At 12 months after immunization, protection from DENV disease and hospitalization were 80% and 95%, respectively, without difference according to age and seropositivity at baseline. Efficacy was highest against DENV2 (97.7%) and lowest against DENV3 (62.6%), although efficacy against DENV4 could not be calculated due to the low incidence of diseases associated with this serotype [40]. Protection slightly declined with time, more significantly in previously unprimed subjects, as evidenced in a study carried out 18 months after vaccination. However, it remained significant against severe DENV requiring hospitalization [41]. After 3 years, global efficacy was still 62.0% against DENV disease and 83.6% against hospitalization with lower values (54.3% against virologically confirmed dengue (VCD) and 77.1% against hospitalization) in baseline seronegative ones compared to baseline seropositive ones (65.0% against VCD and 86.0% against hospitalization). However, analysis of protection against different serotypes showed that the efficacy against hospitalization remained high among seropositive subjects regardless of the infecting serotype but not among seronegative children, as in these subjects, the vaccine was efficacious only against DENV1 and 2. This could suggest the need for a booster dose in these children. Tolerability and safety were very good as incidence of adverse events was low (about 3%) and quite similar between children receiving vaccine and controls, and no serious event was reported [42]. Despite some positive results, variations in vaccine efficacy deserve attention. Moreover, further studies to better evaluate long-term protection and the need for further doses, especially in unprimed children, should be performed. Finally, efficacy and safety in adults and elderly people and in immunocompromised subjects should be evaluated. 

A third DENV vaccine has been evaluated in phase II trials [43]. It is identified as TV003/TV005 and comprises attenuated full-length DENV1, 3, and 4 together with one chimeric virus, in which the prM and E genes of DENV4 are substituted by those of DENV2. Attenuation was obtained by introducing homologous 30-nucleotide deletions into 3′ untranslated regions of DEN1, DEN2, and DEN4. Moreover, 30- and 31-nucleotide deletions were introduced into the 3′ UTR of DEN3. Finally, more mutations in non-structural proteins were added. TV003 and TV005 differ in the dosage of the rDEN2/4Δ30 component (103 PFU for TV003 and 104 PFU for TV005). The vaccine was prepared to have a product able to confer long-term protection against all DENV serotypes with a single dose [43]. Immunological studies seem to indicate that TV003 induces a significant antibody response without relevant adverse events. With a double-blind, randomized, placebo-controlled phase II trial, it has been shown that the administration of a single vaccine dose was associated 90 days later with seroconversion for all four DENV serotypes in most of the vaccinees, regardless of whether they were seropositive or seronegative at baseline. Skin rash was the only relevant adverse event [44]. A phase III clinical trial (NCT02406729) has been planned in Brazil to test the vaccine in subjects aged 2 to 59 years but, despite being active, it is not currently recruiting.

Table 1 summarizes the main available DENV vaccines, which are all based on live attenuated viruses. As highlighted in the table, the limitations of these preparations are significant, which explains why even those authorized in some countries have not been widely used. 

## 3. West Nile Virus Disease

WNV is a zoonotic arthropod-borne flavivirus that encompasses nine lineages, although only lineages 1 and 2 are associated with the development of epidemics. Lineage 1 has a global spread and is responsible for most cases diagnosed in Europe, Africa, and the Americas, including those with severe neurological manifestations [45]. Lineage 2 was exclusively identified in Africa until 2004 and was initially considered less pathogenic than lineage 1 [46]. However, recent reports indicate that this lineage has been introduced into the European Union (Greece, Hungary, and Italy) and Russia [47] and that, like lineage 1, it can cause severe WNV disease [48,49]. Birds are the main reservoir [50] and, when infected, they transmit WNV to mosquitoes of the Culex species, especially Culex pipiens, Culex tarsalis, and Culex quinquefasciatus. These, in turn, spread the virus to humans and other mammals, including horses [51]. Different routes of transmission are exceptional. Cases of vertical transmission and transmission through blood transfusion, breast milk, and organ transplantation have been described [52,53,54]. Generally, mammals are dead-end hosts of WNV as they, although infected after the mosquito bite, do not produce high levels of viremia. Consequently, they do not pass the virus to other biting mosquitoes and do not contribute to the spread of the infection [55].

Clinically, WNV infection can remain totally asymptomatic, cause a flu-like disease generally passing in a few days, or cause severe neurological manifestations such as encephalitis, meningitis, or acute flaccid paralysis [56]. To date, WNV has been diagnosed in every continent except Antarctica generally during summer months and early fall according to mosquito circulation. However, the true incidence of WNV infection in different geographic areas is not precisely defined as available epidemiological data largely underestimate the true incidence of infection. Most WNV infections, 70–80%, are asymptomatic and all these cases are lost. Among the cases that present with flu-like symptoms and account for about 20% of the total, a significant percentage is not identified as specific tests for WNV infection detection are not routinely per-formed. Even among the few WNV infections that occur with severe clinical manifestations (<1%), several are not identified. Clinicians are more likely to test the patient for herpes simplex virus or enterovirus infections rather than for WNV infection. A study carried out in Texas in patients hospitalized for encephalitis or meningitis during the period 2005–2010 has shown that specific tests for WNV infection were performed in only 37% of the cases, even during the WNV season [57]. The difference between the number of WNV cases officially reported and the true incidence in the general population is clearly evidenced by the data collected in the USA. For the period 1999–2010, a total of 12,835 neurological WNV cases were reported [58]. As previous studies have estimated that 30–70 asymptomatic or mild cases of WNV disease occur for every case of WNV neuroinvasive disease, it was calculated that in the study period, a total of 385,050–898,450 non-neuroinvasive disease cases would have been expected to occur. However, only 9034 were officially reported [58]. Despite these limitations, available data clearly indicate that the epidemiology of WNV has significantly changed in the last 30 years and that the disease is endemic in geographic areas such as Europe and a large part of North America, where initially only imported cases were diagnosed. Until the early 1990s, only sporadic cases and outbreaks in Africa, Australia, and the Middle East had been described [59]. From 1996, human outbreaks became more frequent, with increased reporting of severe cases, particularly in the Middle East and Europe [60]. Later, a significant expansion of the European areas affected by WNV progressively occurred and several major outbreaks, such as those described in 2010 and 2018, were documented [61,62]. A further increase in the number of WNV infections was reported in some European countries in 2022 and 2023. As of 30 June 2023, 1133 human cases of WNV infection were reported to the European Surveillance System (TESSy), including 92 deaths for 2022 [63]. Of these, only 20 were not locally acquired, showing the endemicity of the infection. In most of the cases, these WNV infections were diagnosed in Italy (723) and Greece (283), with death reported in 7.1% and 11.6% of the cases, respectively. However, several cases were diagnosed in other Euro-pean countries, showing that WNV infection is endemic in all of central and southern Europe [63]. Similar epidemiological evolution was demonstrated in North America where, after the first evidence of WNV was found in 1999, a very rapid diffusion of the virus across the continent was reported [64]. It has been reported that, in 2021, cases of WNV disease had been diagnosed in every state of the USA. A total of nearly 3000 cases had been identified, a number three times higher than the number of cases diagnosed in 2003. Despite its relatively low virulence in human hosts, WNV is now considered to have the widest geographic distribution and the largest range of vector and host species among all vector-borne flaviviruses, representing an ongoing risk to global health [64].

No therapies for the treatment of WNV disease are presently available. Repeated attempts have been made to use polyclonal immune globulins (IVIGs), polyclonal IVIG with high titers of WNV neutralizing antibodies, a recombinant humanized monoclonal antibody, interferon, corticosteroids, and ribavirin, particularly in patients with proven WNV disease and severe neurological involvement, recipients of solid organ transplants, and immunocompromised patients [65]. However, as no real benefit was evidenced, none of these measures is recommended. 

Several WNV vaccines have been prepared for prevention of WNV infection in animals. They are currently used with satisfactory results, although they require multiple primary doses and an annual booster. Because of these limitations, they are not suitable for use in humans for whom the ideal vaccine should induce a robust long-term protective immunity and require a maximum of one booster dose [66]. Several attempts to develop WNV vaccines have been made, but none of the studied preparations has progressed beyond phase I or II clinical trials. All of the already-cited reasons that generally limit the development of vaccines against mosquito-vectored flavivirus infections explain why the development of a WNV vaccine has not progressed. Even ADE is a potential safety concern, although it has not been observed with WNV infections in people with previous flavivirus exposure. Among the most promising preparations are two live attenuated chimeric vaccines, one DNA vaccine, one recombinant subunit vaccine, and two inactivated whole virus vaccines [67]. Table 2 summarizes data on the main results obtained with WNV vaccine candidates in clinical trials.

ChimeriVax-WN02 is a live attenuated vaccine based on a yellow fever 17D virus in which the pre-membrane and envelope protein genes are replaced with the corresponding genes of the WN NY99 virus [68]. Two phase I (NCT00442169, NCT00442169) clinical trials and one phase II (NCT00746798) clinical trial have shown that a single administration of the vaccine induced seroconversion in >95% of recipients without relevant adverse events with antibody titers and viremia higher among people ≥65 years of age. Similar findings were obtained with three phase I studies (NCT00094718, NCT00537147, NCT0218662) in which two doses of the second live attenuated WNV vaccine, WN/DEN4-3′Δ30, were used. This vaccine is based on chimerization of the wild-type WNV NY99 genome with that of the live attenuated DENV4 candidate vaccine rDEN430. In particular, the genes encoding the prM and envelope proteins of DENV4 were replaced with those of WNV NY99 [69]. 

The DNA vaccine encodes the protein prM and the E glycoproteins of the NY99 strain of WNV under the transcriptional control of the CMV/R promoter [70]. More than 95% of recipients who used this vaccine developed neutralizing antibodies, but this result was obtained only after administration of three doses 4 weeks apart (NCT00300417). Multiple doses are also required to obtain seroconversion in subjects who are given the inactivated whole virus vaccines. Administration of two doses of the hydrogen-peroxide-inactivated WNV vaccine has led to detectable neutralizing anti-bodies in only about 50% of the study participants, leading the authors to conclude that the result might be improved by adding a third dose or by using an alternative in-activation protocol which combines hydrogen peroxide and formaldehyde. Moreover, after two doses and a booster of the formaldehyde inactivated WNV vaccine, high titers of neutralizing antibodies were developed in a satisfactory number of vaccinees. Finally, a recombinant subunit vaccine targeting the E protein was tested in pre-clinical studies and in phase I clinical trials but the results were unsatisfactory [70].

## 4. Chikungunya Virus Disease

CHIKV is an arbovirus belonging to the genus Alphavirus of the Togaviridae family [71]. Four lineages of CHIKV representing different geographic lineages and named as West African (WA) lineage, East/Central/South African (ECSA) lineage, Asian lineage, and Indian Ocean lineage (IOL) are presently circulating in the world. However, as antibodies evoked by a genotype cross-react against the other genotypes, CHIKV exists as a single serotype thought to confer life-long immunity in re-covered subjects. For years, CHIKV has been vectored by mosquitoes of the species Aedes aegypti that are only common in the tropics and subtropics. Outbreaks of CHIKV disease were described in these geographic areas, mainly in Africa and Asia, and only imported cases were documented in Australia, the USA, Canada, and continental Europe [71]. As of 2006, a genetic variation has allowed the virus to be vectored by Aedes albopictus [72]. As this species of mosquito is endemic almost globally, CHIKV disease could occur worldwide [73]. In 2007, an outbreak of autochthonous CHIKV infections took place for the first time in continental Europe. Between July and September in Emilia-Romagna in the northeast of Italy, 217 cases of CHIKV infection were diagnosed [74]. In 2013, CHIKV was found in the Caribbean and quickly spread to the Americas, particularly the southern countries. In June 2014, the first locally acquired case of CHIKV in Florida, USA, was confirmed, and a total of 272 imported cases and 11 locally acquired cases had been reported there by October 14, 2014 [75]. At present, infection is endemic in more than 100 countries. In 2023, as of 23 August, approximately 320,000 cases and over 340 deaths were reported worldwide [76]. However, it seems likely that the total number of cases that occur annually worldwide is significantly greater. Up to more than 20% of CHIKV infections remain asymptomatic [77]. Many patients exhibit signs and symptoms quite similar to several other viral infections, including dengue and Zika, and as a result are not identified. Finally, even when CHIKV is strongly suspected, confirmation can be difficult in low- and middle-income countries where expensive laboratory tests for virus identification, such as reverse transcriptase polymerase chain reaction (RT-PCR), are not available due to cost.

The disease is characterized by an abrupt onset of fever, frequently accompanied by joint pain and, to a lesser extent, joint swelling, muscle pain, headache, nausea, fatigue, and rash. Most patients recover fully from the infection that leaves a permanent immunity from future infections [78]; however, several patients present debilitating joint pain for months or years after the disease onset. Finally, occasional cases of severe disease with eye, heart, and neurological complications have been reported. Neonates infected during delivery, elderly people, and subjects with underlying chronic severe diseases are at increased risk of death [79]. 

Against CHIKV infection, neither specific treatment nor vaccines are available. However, several vaccines are currently in development involving a large array of technology platforms. Studies on inactivated whole virus vaccines, live attenuated vaccines, genetically engineered vaccines including subunit vaccines, virus-vectored vaccines, virus-like particle-based vaccines, and nucleic acid vaccines have been re-ported [80]. In some cases, despite promising results, development was abandoned. This is the case for a live attenuated vaccine obtained from a serially passaged, plaque-purified live CHIKV strain derived from an infected patient. A clinical study revealed that the preparation was safe and well tolerated and that 98% and 85% of vaccinees had neutralizing antibodies 28 days and 1 year after immunization, respectively. However, no further studies after the year 2000 were carried out [81]. Other vaccines are far behind in their development or have limitations. 

An inactivated vaccine (BBV87), formulated with an inactivated whole virus, based on the East/Central/South African genotype strain was found to be able to generate satisfactory short- and long-term immune responses and was considered for phase II and III clinical trials in adults. However, the trials (NCT04566484) are active but not recruiting. Moreover, the schedule of administration includes two doses, which makes the preparation less attractive compared with vaccines able to evoke a similar immune response with only one dose.

The recombinant chimpanzee adenovirus-vectored vaccine (ChAdOx1-Chik) containing the full-length polyprotein of CHIKV has proven promising in a phase I trial because of its ability to induce an early significant specific immune response without adverse events [82]. However, no further development was reported. It cannot be ruled out that this could, at least in part, depend on the concerns regarding the safety of adenovirus-vectored vaccines, developed after the adverse events reported with the adenovirus-vectored SARS-CoV-2 vaccine [83]. 

MV-CHIK, a measles-vectored, live attenuated, recombinant vaccine, was found to be strongly immunogenic and protective in animal studies and showed good safety, tolerability, and immunogenicity in phase I and II clinical trials, although to obtain persistence of significant specific antibody levels, two doses of the vaccine are required. No study on this is currently in progress [84].

Several mRNA vaccines have been studied but all of them are in a very early phase of development. Moreover, even with these vaccines, the best results were obtained with two vaccine doses. An mRNA-lipid nanoparticle (mRNA-LNP) vaccine expressing CHIKV E2-E1 antigen was shown to be highly immunogenic in animals when ad-ministered with a prime booster schedule [85]. Another vaccine candidate consists of two RNAs: a non-replicating mRNA encoding for the CHIKV non-structural proteins, and a trans-amplifying RNA (taRNA) encoding the CHIKV envelope proteins. This allows a potent immune response with small amounts of mRNA as found in experimental animals and in one phase I trial in humans [86]. Finally, an mRNA vaccine in which mRNA (mRNA-1944) was used to deliver a monoclonal, CHIKV-specific, neutralizing antibody has been developed. A first phase I clinical trial has shown that this vaccine can induce seroconversion and seroprotective antibody concentrations without significant adverse events, with the best immune response being demonstrated when two doses were administered [86]. 

The results obtained with CHIKV VLP, an adjuvanted virus-like particle (VLP)-based vaccine, are very interesting. It has been investigated with two phase III randomized, double-blind, placebo-controlled studies involving subjects aged 12 to 64 years (NCT05072080) and people over 65 years (NCT05349617). A strong specific antibody production was shown in most of the participants within a couple of weeks from injection. Moreover, data collected in adolescents and adults indicate that sero-protective antibody levels could be demonstrated in 86% of vaccinees 6 months after vaccination. Regarding safety, CHIKV VLP was well tolerated, with adverse events being mostly mild or moderate in nature [87]. 

However, the only vaccine for which results of a phase III trial have been published is VLA 1553 [88]. This is a live attenuated vaccine based on the East/Central/South African virus genotype in which attenuation is obtained through a 61 amino acid deletion in non-structural protein 3. After identification of the vaccine dose capable of inducing seroconversion in all vaccinated adults without relevant ad-verse events [89], the potential efficacy of this preparation was tested in a double-blind, multicenter, randomized trial enrolling healthy volunteers aged 18 years and older. As efficacy trials were considered unfeasible due to the unpredictability of CHIKV epidemiology and the poor efficacy of many national virus circulation surveillance systems [90], it was decided to evaluate the vaccine considering its ability to induce an immune surrogate of protection defined as a CHIKV-specific neutralizing antibody titer of ≥150 [91]. In this study, a total of 3093 participants were enrolled (3093 VLA1553 and 1035 placebo), and among them, 266 in the VLA1553 group and 96 in the placebo group were tested for immunogenicity. The results showed that a single dose of VLA1553 was able to evoke a seroprotective immune response in 98.9% of participants in the VLA1553 group (95% confidence interval (CI) 96.7–99.8; *p* < 0.0001) 28 days post-vaccination, independently of age. Although slightly lower than 1 month after vaccine administration, specific antibody levels remained significantly higher than the putative level of protection at 3 and 6 months after vaccine administration, suggesting long-term protection. Monitoring of adverse events revealed that VLA1553 was generally safe. Serious adverse events were reported in 1.5% of 3082 participants receiving VLA1553 and in 0.8% of those given the placebo. Only two serious adverse events were considered related to VLA1553 treatment. Based on these results, on 9 November 2023, the U.S. FDA approved this vaccine for individuals aged 18 and older at increased risk of exposure to CHIKV [92].

Table 3 summarizes the main available CHIKV vaccines, highlighting limitations of their use.

## 5. Problems in Development of Further Vaccines against Emerging Mosquito-Vectored Arbovirus Infections

Regarding other infections, in addition to the prevention of DENV, WNV, and CHIKV infection, an ideal vaccine should be able to evoke with a single administration a prompt activation of B and T cells, the production of adequate concentrations of protecting/neutralizing antibodies, and the creation of a strong immune memory capable of triggering an effective secondary antibody response after new infection with a wild-type and/or mutated infectious agent [93,94]. Moreover, to establish efficacy, re-liable data collected through adequate randomized, double-blind, placebo-controlled clinical trials involving large numbers of people of different ages should be obtained. Incidence of the disease in vaccinated people and controls should be carefully evaluated in order to analyze short- and long-term protection induced by the vaccine. The correspondence between immunological data and real protective capacity in cases of infection should be studied. Finally, the vaccine should be well tolerated, safe, easily administrated, cost-effective, and widely available throughout the world [95]. However, achieving these goals is not easy, particularly when mosquito-vectored arbovirus infections are considered. 

## 6. Conclusions

Preparing a phase III trial is frequently difficult due to the sporadic and unpredictable nature of arbovirus disease outbreaks. Moreover, evaluation of vaccine efficacy is not easy as the identification of infected subjects can be very hard because viremia is short-lived and detection of specific antibodies does not allow differentiation be-tween actual infected patients and simple vaccinated subjects. The evaluation of tolerability and safety, especially when considering relatively uncommon but clinically very relevant adverse events, is made difficult by the need for the enrollment of a great number of subjects. Finally, costs can be prohibitive. All these limitations explain why the development of vaccines against the emerging arbovirus diseases is far from being satisfactory, and it seems likely that it will take many years before effective and safe vaccines for all these infections are made available worldwide. To overcome these problems and simplify the evaluation of vaccine potential efficacy, it has been suggested that new vaccines should be tested simply by analyzing the immune response and the achievement of antibody levels higher than the minimum correlate of protection [96]. This method has already been used for the CHIKV vaccine and it will likely be used for other arbovirus vaccines. Instead of measuring the reduction in the incidence of the disease in vaccinated subjects compared to unvaccinated controls, evaluation of the presence of a supposed protective antibody level can significantly reduce complications of clinical trials. The use of recombinant genetic technology, such as in the case of DNA and RNA vaccines, replicon vaccines, and vaccines with epitope modification, can enable the development of more effective vaccines in a shorter time. Thus, the problem of the availability of effective and safe vaccines against DENV, WNV, and CHIKV infections can be more rapidly solved [97]. The activities of organizations such as the Coalition for Epidemic Preparedness Innovations (CEPI) can significantly accelerate the development and approval of new effective and safe vaccines against all arbovirus infections. This is an innovative global partnership between public, private, philanthropic, and civil society organizations, which are working together to finance and coordinate the development of new vaccines and prevent and contain infectious disease epidemics [98]. 

## Figures and Tables

**Table 1:** Main available dengue virus (DENV) vaccines.

Candidate Name	Main Limitations
Dengvaxia	Poorly effective with an increased risk of severe DENV disease in the case of infection in seronegative children, low efficacy in younger children, high cost, and the need for three doses to obtain protection
Qdenga	Variations in vaccine efficacy; few data on long-term protection, especially in unprimed children; lack of information on efficacy and safety in adults, elderly people, and immuno-compromised subjects
TV003/TV005	Phase III trials are ongoing

**Table 2:** West Nile virus vaccine candidates tested in clinical trials.

Vaccine Type, Name and Trial	Age of Participants (yr)	No. of Participants Enrolled	No. and Timing of Doses	Immunogenicity	Serious Adverse Events Attributed to Vaccine	ClinicalTrials.gov Number
**Live Attenuated Chimeric**						
**WN/DEN4-3′630 (NIAID, NIH)**						
Phase I trial, completed in 2005	18–50	56	1 dose	55–75% seroconversion (increase in PRNT_50_ by a factor of ≥4 at day 28 or 42)	None	NCT00094718
Phase I trial, completed in 2009	18–50	26	2 doses, 6 mo apart	89% seroconversion after second dose (increase in PRNT_50_ by a factor of ≥4 at day 28 or 42)	None	NCTOOSJ7147
Phase I trial, completed in 2016	50–65	28	2 doses, 6 mo apart	95% seroconversion after fìrst dose by day 90 (PRNT_50_ ≥ 1:10)	None	NCT02186626
**ChimeriVax-WN02, YFV, 17D backbone (Sanofi-Pasteur)**						
Phase II trial, completed in 2009						
Part I	18–40	112	1 dose	>96% seroconversion (increase in PRNT_50_ by a factor ≥4 at day 28)	None	NCT00442169
Part II	≥41	96	1 dose	Approximately 96% seroconversion (increase in PRNT_50_ by a factor of ≥4 at day 28); GMT and viremia tended to be higher among people >65 yr of age	None	NCT00442169
Phase II trial, completed in 2009	≥50	479	1 dose	92–95% seroconversion (increase in PRNT_50_ by a factor ≥4 at day 28)	None	NCT00746798
**DNA**						
**VRC-WNVDNA0l7-00-VP, first generation (NIAID, NIH, DVBD, CDC)**						
Phase I trial, completed in 2008	18–50	15	3 doses 4 wk apart	100% had neutralizing antibodies detected (PRNT_50_) at 4 wk and 24 wk after third dose; titers were similar to those elicited in horses 3 wk after 1 dose of WNV DNA vaccine	None	NCTOOI06769
**VRC-WNVDNA020-00-VP, second generation (NIAID, NIH)**						
Phase I trial, completed in 2007	18–65	30	3 doses 4 wk apart	>96% had neutralizing antibodies detected (EC_50_) at 4 wk after third dose	None	NCT00300417
**Recombinant subunit**						
**WN-80E (Hawaii Biotech)**						
Phase I trial, completed in 2009	18–45	25	3 doses 4 wk apart	Unpublished	Unpublished	NCT00707642
**Inactivated whole virus**						
**HydroVax-001, hydrogen peroxide inactivated (Najit Technologies)**						
Phase I trial, completed in 2016	18–49	51	2 doses (1 µg or 4 µg), 4 wk apart	31–50% seroconversion at 15 days after second dose (PRNT_50_ ≥ 20 with or without complement enhancement)	None	NCT02337868
**Formalin inactivated (Nanotherapeutics)**						
Phase I–II trial, completion date unknown	≥18	320	2 doses 3 wk apart; booster on day 180	All doses elicited immune response, with peak antibody responses after booster dose (microneutralization)	None	None

**Table 3:** Main available Chikungunya virus (CHIKV) vaccines.

Vaccine Type	Candidate Name	Main Limitations
Inactivated vaccine	BBV87	Active trials but without enrollment, requires two doses
Recombinant chimpanzee adenovirus-vectored vaccine	ChAdOx1-Chik	No further development after phase I trial because of concerns regarding safety of adenovirus-vectored vaccines
Measles-vectored, live-attenuated, recombinant vaccine	MV-CHIK	No further development after phase II trials, requires two doses
mRNA vaccines	mRNA-LNP taRNA mRNA-1944	Only phase I data available
Adjuvanted virus-like particle-based vaccine	CHIKV VLP	Strong specific antibody pro-duction and good safety profile in adolescents, adults, and the elderly
Live attenuated vaccine	VLA 1553	Strong immunogenicity and good safety in individuals aged 18 years and older at increased risk of exposure to CHIKV

## Data Availability

Not applicable.
